# Spanish Translation and Psychometric Evidence of the Nightmare Disorder Index (NDI) in Adults from the General Population

**DOI:** 10.3390/ejihpe15110220

**Published:** 2025-10-27

**Authors:** Jonatan Baños-Chaparro, Andrei Franco-Jimenez, Tomás Caycho-Rodríguez, Diego Valencia-Pecho, Esteban Sarmiento-Suarez, Dulce Bernabel-Tarazona, Gabriela Rivera-Álvarez

**Affiliations:** 1 Programa Académico de Psicología, Facultad de Ciencias de la Salud, Universidad Privada Norbert Wiener, Lima 15046, Peru; diego.valencia@uwiener.edu.pe (D.V.-P.); a2023102054@uwiener.edu.pe (E.S.-S.); a2024102782@uwiener.edu.pe (D.B.-T.); a2023102380@uwiener.edu.pe (G.R.-Á.); 2 Facultad de Psicología, Universidad Nacional San Luis Gonzaga, Ica 11000, Peru; andrei.franco@unica.edu.pe; 3 Facultad de Psicología, Universidad Científica del Sur, Lima 15046, Peru; tcaycho@cientifica.edu.pe

**Keywords:** sleep disorders, nightmares, mental health, adult, translation

## Abstract

Background: Nightmares are a type of sleep disorder characterised by vivid and distressing dreams that cause abrupt awakenings, leading to significant discomfort. In adults, recurrent nightmares can negatively impact quality of life, daytime functioning, and overall mental health. In this context, it is essential to have valid, reliable, and culturally appropriate psychological instruments that allow for an accurate assessment of this phenomenon. The aim of the present study was to translate and validate the Nightmare Disorder Index (NDI) into Spanish for use with Peruvian adults. Methods: A total of 507 adults (66.7% women) participated by completing a sociodemographic questionnaire and psychological instruments. Statistical analyses were conducted using structural equation modelling and item response theory. Results: The NDI demonstrated adequate content validity (V > 0.70), a unidimensional structure (CFI = 0.99, RMSEA = 0.06 [90% CI: 0.030, 0.102], SRMR = 0.03), and reliability (ω = 0.84, H = 0.94, *r*_xx_ = 0.79). In addition, invariance was observed across sex, and significant associations were found with depressive symptoms, generalised anxiety, and suicidal ideation. Item 3 showed the highest discrimination and information, and the scale proved to be accurate at higher levels of nightmare severity. Conclusions: The NDI presents adequate psychometric properties for the inference and interpretation of scores in the assessment of nightmares. Its use is recommended in both professional practice and research with the adult general population.

## 1. Introduction

Nightmares are vivid and disturbing experiences that disrupt sleep, reduce its restorative quality, and often involve themes of physical aggression, failure, or helplessness (Robert & Zadra, 2014; Sheaves et al., 2023). They are also associated with poorer sleep quality, reduced subjective wellbeing, and an increased risk of cardiovascular problems (Blagrove et al., 2004; A. A. Campbell et al., 2023; Simor et al., 2012). From an epidemiological perspective, the figures are striking: up to 30% of psychiatric patients present with significant problems related to nightmares, and in the general population around 1 in 20 individuals experience weekly nightmares (Li et al., 2010; Swart et al., 2013). In Latin America, findings are similar, as sleep disorders in general affect approximately 25% of the population (Etindele Sosso et al., 2023). In this regard, when nightmares are recurrent and interfere with daily activities, they may meet the criteria for nightmare disorder (American Psychiatric Association, 2022).

Despite their clinical relevance, nightmare disorder often remains underdiagnosed and undertreated, partly because many clinicians are insufficiently informed about its consequences or about effective therapeutic options (Nadorff et al., 2015). In routine practice, patients rarely mention nightmares unless clinicians inquire directly, and systematic screening is uncommon. These factors contribute to these symptoms being overlooked in favour of comorbid problems and to the misconception that nightmares resolve on their own (Thünker et al., 2014). As a result, the disorder continues to be underestimated in both clinical and research settings, which limits recognition and timely intervention.

In the adult general population, nightmares may appear independently or as a symptom associated with a range of mental health problems, such as post-traumatic stress disorder, schizophrenia, bipolar disorder, insomnia, depressive symptoms, anxiety disorders, and suicidal ideation—well-established risk factors for suicidal behaviour (Akkaoui et al., 2020; Baños-Chaparro et al., 2024; R. L. Campbell & Germain, 2016; Delage et al., 2024; Sandman et al., 2017; Sheaves et al., 2023; Swart et al., 2013). Collectively, these findings highlight nightmares as a clinically relevant phenomenon in mental health, both as an independent condition and in comorbidity with other psychological disorders.

Nevertheless, nightmares often go unnoticed in clinical practice, even though effective treatments are available. For instance, Imagery Rehearsal Therapy is the first-line treatment and has been shown to reduce the frequency, severity, and distress associated with nightmares (Gill et al., 2023). Other behavioural interventions, such as exposure and relaxation (Morgenthaler et al., 2018), as well as pharmacological options (Skeie-Larsen et al., 2022), have also demonstrated efficacy. Historically, nightmares have not been a central focus of behavioural therapy and were often regarded as a secondary symptom expected to disappear once other disorders were treated (Thünker & Pietrowsky, 2012). This historical under-recognition may be partly due to the absence of validated assessment tools, which makes it difficult to recognise nightmare disorder as an independent condition. The availability of a validated instrument would allow for the systematic detection of nightmare disorder, recognition as an independent condition, and timely access to evidence-based therapies.

Furthermore, research has shown that nightmares differ by sex in the adult general population. In terms of prevalence, women tend to report nightmares more frequently than men, a phenomenon associated with both biological factors (such as hormonal differences and sleep architecture) and psychosocial factors (e.g., higher levels of negative affect or emotional vulnerability) (Cappadona et al., 2021; Dobler et al., 2025; McNamara et al., 2025). Some studies also suggest that women experience dream content characterised by greater fear, anxiety, and sadness, whereas in men nightmares are more often linked to themes of aggression or external threat (Cappadona et al., 2021; Dobler et al., 2025). These differences highlight the need to examine measurement invariance in instruments assessing nightmares to ensure that scores are equivalent across sexes and comparisons are psychometrically valid.

Although several instruments exist to assess nightmare disorder, many omit key diagnostic criteria. For example, the Disturbing Dreams and Nightmare Severity Index does not include symptom chronicity or dream recall upon awakening (Krakow et al., 2002); the Nightmare Distress Questionnaire does not consider frequency, chronicity, or recall (Belicki, 1992); and the Nightmare Experience Questionnaire does not include frequency or chronicity (W. Chen et al., 2014). To address this gap, the Nightmare Disorder Index (NDI) was developed a five-item self-report measure aligned with DSM-5 criteria, which assesses frequency, chronicity, dream recall, and the associated distress and impairment. During its initial validation, the NDI showed good internal consistency (*α* = 0.80), a unidimensional factor structure (eigenvalue = 2.31; 33.7% variance), and adequate construct validity, with moderate correlations with nightmare frequency and severity measures from sleep diaries (Dietch et al., 2021). A subsequent Chinese validation also reported high internal consistency (*α* = 0.88) and two-week test–retest reliability (Zhuang et al., 2023). However, it has not yet been translated into Spanish, which limits its applicability in Spanish-speaking populations and underscores the need for a translated and validated version for research and clinical use.

In the Peruvian context, empirical evidence on nightmares is scarce, and no study has validated a DSM-5–based instrument for nightmare disorder in adults. Validating the NDI in this population would facilitate the systematic assessment of nightmares and support its clinical application. Furthermore, given that the experience of nightmares may vary according to cultural context, its validation in the Peruvian population is particularly relevant (Hollan, 2009).

For these reasons, the objective of this study was to translate the NDI into Spanish and evaluate its psychometric properties in Peruvian adults from the general population. Specifically, it was hypothesised that (a) the NDI would show a unidimensional factor structure consistent with previous studies; (b) the scale would demonstrate adequate reliability; (c) measurement invariance would hold across sex; (d) NDI scores would correlate positively with depressive symptoms, generalised anxiety, and suicidal ideation, providing evidence of validity based on relationships with other variables; and (e) item response theory analyses would support adequate discrimination, difficulty, and information functions of the items.

## 2. Materials and Methods

### 2.1. Participants

This study adopted an associative, quantitative approach with a cross-sectional design. Data were collected through an online survey administered between January and April 2025 using the Google Forms platform. The questionnaire was disseminated via the researchers’ social networks (Facebook y WhatsApp) and included information about the purpose of the study, the anonymity of responses, their academic use, data management, and the obtaining of informed consent. Digital surveys facilitate broader reach to the target population, allow systematic control of responses, provide multiple dissemination channels, and represent an effective and low-cost alternative for implementation (Gosling & Mason, 2015). The participants did not receive financial compensation.

In total, 507 Peruvian adults from the general population participated. The majority were women (66.7%) with a mean age of 33 years (SD = 12.05; range 18–60 years). In addition, 72.4% reported being employed at the time of the survey, while 27.6% were unemployed. Regarding marital status, most identified as single (72%) or married (25%), with smaller proportions of divorced (2.8%) and widowed (0.2%) participants. The vast majority resided in urban areas (95.9%). In terms of educational attainment, the largest group were participants with incomplete university studies (32.7%), followed by those with completed university studies (26.2%) and completed technical education (12.2%).

### 2.2. Measures

#### 2.2.1. Demographic Information

A brief form was used to collect information from participants, which included questions on gender, age, employment status, marital status, area of residence, and level of education.

#### 2.2.2. Nightmare Disorder Index (NDI)

The NDI is a brief instrument consisting of five items designed to assess the symptoms of nightmare disorder in accordance with DSM-5 diagnostic criteria. The NDI items evaluate the frequency and characteristics of nightmares, as well as the distress and impairment associated with them over the past month, with scores ranging from 0 to 4. The total score ranges from 0 to 20, where higher scores indicate greater severity of nightmare disorder (Dietch et al., 2021).

The back-translation method was employed to adapt the instrument into Spanish. In the first stage, a professional bilingual translator independently translated the scale from English into Spanish. A second translator then carried out the back-translation, from Spanish into English. Subsequently, a review meeting was held between both translators and the research team to reach consensus and approve the final version. Thereafter, five expert judges in clinical and educational psychology were invited to evaluate the items according to three fundamental criteria: relevance, representativeness, and clarity. Finally, prior to formal administration, a pilot test was conducted with seven adults to assess comprehension and clarity of the items in the target population. No suggestions for modification were received at this stage. Table 1 presents both the original English version and the Spanish translation.

#### 2.2.3. Frequency of Suicidal Ideation Inventory (FSII)

This inventory assesses the frequency of suicidal ideation over the past year using five items. Items are rated on a Likert scale ranging from 1 (never) to 5 (almost every day). The total score ranges from 5 to 25, with higher scores indicating greater frequency of suicidal ideation. The adapted Peruvian version was used, and this study found acceptable reliability (ω = 0.96) (Baños-Chaparro et al., 2021b).

#### 2.2.4. Patient Health Questionnaire-2 (PHQ-2)

The PHQ-2 is a brief two-item questionnaire that assesses depressive symptoms over the past two weeks. Each item is rated on a four-point scale from 0 (not at all) to 3 (nearly every day). The total score ranges from 0 to 6, with higher scores indicating greater depressive symptomatology. The Peruvian adaptation was used, and this study reported good reliability (ω = 0.73) (Baños-Chaparro et al., 2021a).

#### 2.2.5. Generalised Anxiety Disorder-2 (GAD-2)

The GAD-2 is a short scale assessing generalised anxiety over the past two weeks through two items. Each item is rated on a four-point scale from 0 (not at all) to 3 (nearly every day). The total score ranges from 0 to 6, with higher scores reflecting greater levels of generalised anxiety. The Peruvian adaptation was used, and this study reported adequate reliability (ω = 0.88) (Baños-Chaparro, 2022).

### 2.3. Statistical Analysis

The statistical analysis was conducted in several stages using RStudio software (version 4.3.2). In the first stage, a descriptive analysis of the items was carried out, considering measures such as mean, standard deviation, skewness, kurtosis, polychoric correlation matrix, corrected item–test correlations (*r*_it_ > 0.30), and Aiken’s V coefficient (V > 0.70) (Kline, 2023; Roebianto et al., 2023). In the second stage, a confirmatory factor analysis (CFA) was performed using the robust weighted least squares estimator adjusted for mean and variance (WLSMV). To evaluate model fit, indicators such as the Comparative Fit Index (CFI > 0.95), Root Mean Square Error of Approximation (RMSEA < 0.08), and Standardised Root Mean Square Residual (SRMR < 0.080) were used, in addition to examining the standardised factor loadings (ƛ > 0.30) (Jordan Muiños, 2021).

The third stage involved estimating reliability through the Bayesian omega coefficient (ω), H coefficient, and empirical reliability (*r*_xx_) (Baños-Chaparro & Caycho-Rodríguez, 2024; Du Toit, 2003; Gunnell, 2020). In the fourth stage, factorial invariance by sex was assessed using the WLSMV estimator. Initially, a configural model was tested to examine the equivalence of the NDI factor structure between women and men. Subsequently, progressively more restrictive models were specified to test equality between groups at the following levels: thresholds (threshold invariance), factor loadings (metric invariance), intercepts (scalar invariance), and residuals (strict invariance). Model evaluation employed minimum difference cut-off criteria: ΔCFI < 0.010, ΔTLI ≤ 0.010, and ΔSRMR ≤ 0.030 (F. F. Chen, 2007; Finch & French, 2018).

The fifth stage consisted of estimating a covariance-based structural equation model (CB-SEM) to analyse the relationships among depressive symptoms, generalised anxiety, and suicidal ideation variables. Again, the WLSMV estimator was used, with fit assessed through indices such as CFI > 0.95, RMSEA < 0.08, and SRMR < 0.08 (Jordan Muiños, 2021). To interpret the strength of correlations, the following cut-offs were applied: small = 0.10, moderate = 0.30, and large = 0.50 (Cohen, 1988).

Finally, in the sixth stage, a two-parameter IRT model (2PL) was estimated. This considered the discrimination parameter (a), which differentiates whether a person has low or high trait ability (θ), where values > 1 are acceptable (Baker & Kim, 2017). The second parameter was difficulty (β), representing the probability of endorsing one response option over another along the θ continuum. The model was based on the graded response model (GRM), and assumptions were tested, including unidimensionality, local independence using the standardised LD-_X_^2^ statistic (LD-_X_^2^ < 10), and monotonicity via a non-parametric Mokken IRT model with calculation of the crit statistic (crit < 0.40) (Sijtsma & van der Ark, 2017; Stover et al., 2019). Furthermore, information was estimated using item information curves (IIC) and test information curves (TIC), as well as model fit at the item level, assessed with the S-_X_^2^ index and RMSEA.S-_X_^2^ < 0.089 (Maydeu-Olivares & Joe, 2014; Monroe & Cai, 2015). The statistical analysis script can be found in the following open-access repository: https://osf.io/e8g9w/files/osfstorage (accessed on 8 October 2025).

### 2.4. Ethical Considerations

The research adhered to international and national ethical standards in the field of psychology. Participants signed and provided informed consent. The survey was anonymous and voluntary, and data protection was ensured with full confidentiality (Hilbig et al., 2022). In addition, the study was reviewed and approved by the ethics committee of the Universidad Privada Norbert Wiener, under registration No. 0833-2024-CIEIC-UPNW.

## 3. Results

### 3.1. Evidence Based on Content

The expert judges evaluated the content of the items according to the criteria of relevance, representativeness, and clarity, where the Aiken’s V values were higher than 0.70 (Table 2). All judges approved the final Spanish version of the NDI.

### 3.2. Descriptive Analysis

In Table 3, it can be observed that the highest arithmetic mean was found in item 2 (M = 0.94) and the lowest in item 4 (M = 0.43). As for the standard deviation, the highest value was observed in item 5 (SD = 1.27) and the lowest in item 4 (SD = 0.78). Skewness and kurtosis were within the ±2 range, although item 4 slightly exceeded this range. Regarding the corrected item–test correlation, all values were adequate and above 0.30, whereas in the polychoric correlation matrix the relationships were positive, and no evidence of multicollinearity was found (r > 0.90).

### 3.3. Evidence Based on Internal Structure

**Figure 1:**
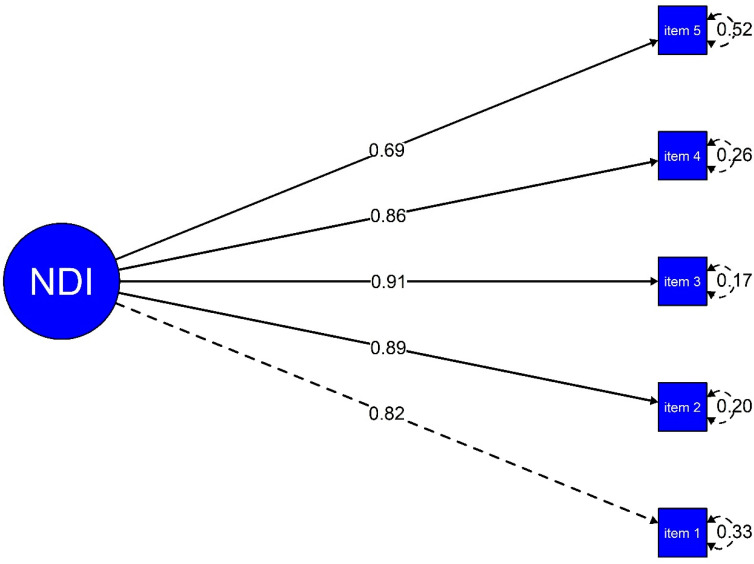
Factor structure of the NDI.

The unidimensional factorial structure of the NDI showed adequate fit indices (CFI = 0.99, RMSEA = 0.06 [90% CI: 0.030, 0.102], and SRMR = 0.03). Moreover, the standardised factor loadings ranged between 0.69 and 0.91, suggesting that all items contribute substantially to the latent construct (Figure 1).

### 3.4. Reliability

The posterior estimate of the ω coefficient was 0.84, with a 95% credibility interval ranging from 0.82 to 0.86, indicating a 95% probability that the true value of ω lies within this range. Likewise, the reliability coefficients H = 0.94 and *r*_xx_ = 0.79 were considered adequate.

### 3.5. Measurement Invariance

Table 4 presents the results of the factorial invariance analysis. It can be observed that the configural model does not differ significantly from the more restrictive models, that is, those imposing constraints on factor loadings, thresholds, intercepts, and residuals, since the changes in the fit indices (ΔCFI < 0.010; ΔTLI ≤ 0.010, ΔRSMR < 0.030) remain within acceptable limits. These results support the invariance of the NDI for women and men.

### 3.6. Evidence Based on the Relationship with Other Variables

**Figure 2:**
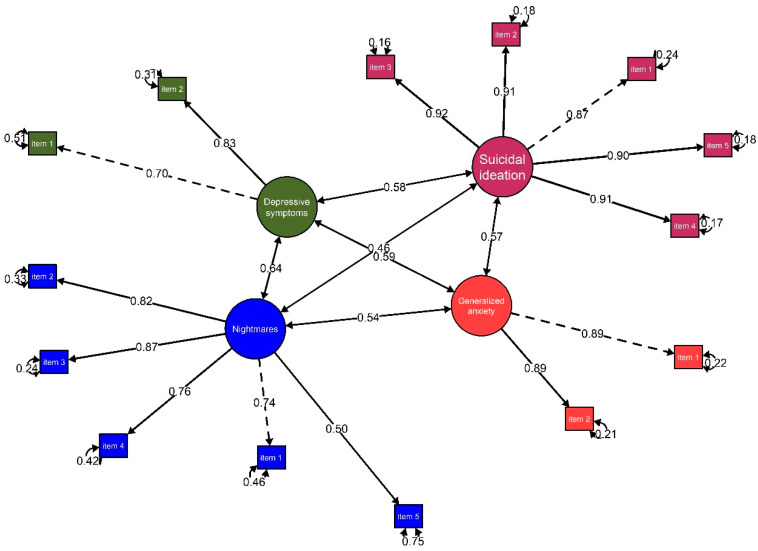
Structural model of the relationship between nightmares, depressive symptoms, generalised anxiety, and suicidal ideation.

Figure 2 presents the correlation analysis between nightmares and other psychological variables. The structural model showed a good fit to the data, with indices including CFI = 0.97, RMSEA = 0.05 [90% CI: 0.040, 0.056], and SRMR = 0.03. It was observed that nightmares maintained positive, statistically significant relationships of strong to moderate effect sizes with depressive symptoms (*r* = 0.64, *p* = 0.001), generalised anxiety (*r* = 0.54, *p* = 0.001), and suicidal ideation (*r* = 0.46, *p* = 0.001).

### 3.7. Item Response Theory

The model assumptions were evaluated and reviewed. Unidimensionality was supported by the CFA, while local independence was confirmed through the LD-_X_^2^ index, with standardised relationships below 10 (values ranged from −0.177 to 0.175). With regard to monotonicity, no significant violations were identified (*crit* < 0.40). In addition, the model fit at the item level was optimal (RMSEA.S-x^2^ < 0.089). Regarding the discrimination parameter (a), item 3 showed the highest ability to differentiate levels of the latent trait (θ). Concerning the difficulty parameter (β), a progressive increase was observed in the probability of selecting higher response options as the level of θ increased, as detailed in Table 5.

**Figure 3:**
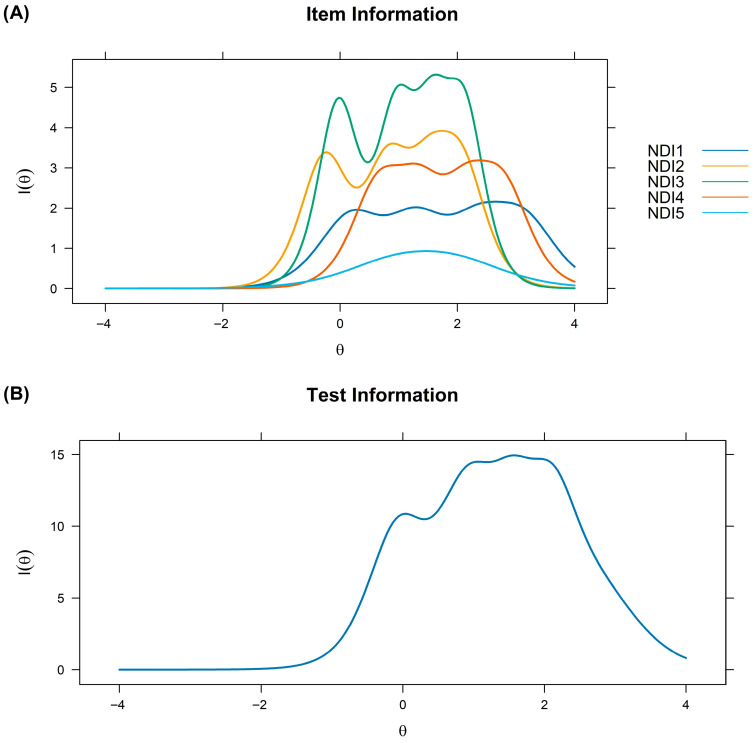
Item information function (**A**) and scale (**B**).

On the other hand, Figure 3 shows that item 3 contributed the greatest amount of information, further highlighting that the scale is particularly accurate for assessing higher levels of θ.

## 4. Discussion

Nightmares constitute a clinically relevant phenomenon that is associated with a wide range of psychological distress and functional impairment. Although they are often considered common experiences, in some cases they reach a frequency and intensity that significantly interferes with daily life, representing a risk factor for mental health problems (Cappadona et al., 2021; Dobler et al., 2025). Having validated and reliable instruments for the general population is essential for the early identification of individuals at risk and for the development of appropriate interventions. In this context, the aim of the study was to translate the NDI into Spanish and validate it in the general adult population, thereby providing a psychometrically robust tool, especially in countries such as Peru, where there remains a scarcity of specific instruments for the assessment of nightmares.

With regard to the evidence based on internal structure, the results supported a unidimensional structure of the NDI, which is consistent with previous findings from the United States and China, both of which also reported a unidimensional structure. This suggests that nightmares can be assessed as a unidimensional construct through the NDI, which reflects the severity and subjective impact of the phenomenon (Dietch et al., 2021; Zhuang et al., 2023). This finding reinforces the utility of the NDI as a parsimonious measure, facilitating its use in both research and clinical practice. Likewise, adequate reliability was observed, supported by the Bayesian omega coefficient, the H coefficient, and the rxx derived from IRT. These indicators suggest that the items of the NDI are consistent and accurately capture the construct of interest (Dietch et al., 2021; Zhuang et al., 2023). The use of more robust indices, such as Bayesian omega and the H coefficient, strengthens the evidence for internal consistency and positions the NDI as a reliable measure for the assessment of nightmares in the study population.

Moreover, the results confirmed measurement invariance of the NDI by sex. Although psychometric studies conducted in the United States and China did not perform this analysis, the findings imply that men and women respond equivalently to the NDI items, ensuring the validity of between-group comparisons (Dietch et al., 2021; Zhuang et al., 2023). The absence of sex bias in measurement is consistent with research showing that, although the frequency of nightmares may vary according to sociodemographic characteristics, the construct measured by the NDI retains the same structure and meaning across different subgroups, reinforcing the applicability of the instrument.

In terms of evidence based on relationships with other variables, it was found that nightmares were positively and statistically significantly associated with depressive symptoms, generalised anxiety, and suicidal ideation. This pattern is consistent with previous psychometric studies and the scientific literature, which indicate that nightmares represent a transdiagnostic feature of psychopathology and a predictor of suicide risk (Akkaoui et al., 2020; Baños-Chaparro et al., 2024; Dietch et al., 2021; Zhuang et al., 2023). A likely explanation is that nightmares disrupt sleep, thereby increasing emotional vulnerability, reducing coping resources, and amplifying anxiety, depressive symptoms, and suicidal behaviours (Sandman et al., 2017; Sheaves et al., 2023). In this regard, the findings emphasise the clinical relevance of systematically assessing nightmares in adults from the general population, as they may help to identify individuals at greater risk of psychological impairment.

From an IRT perspective, all items showed adequate discrimination, and the probability of response across the available options increased progressively from lower to higher levels of difficulty. In the item information curves, item 3 (“To what extent have nightmares generally worried or distressed you?”) contributed the greatest amount of information. This result indicates that the subjective perception of distress associated with nightmares constitutes a relevant indicator, suggesting that researchers and clinicians should pay particular attention to responses to this item (Sheaves et al., 2023). Furthermore, it was observed that the scale provides greater precision in the assessment of high levels of nightmares, which is clinically relevant for identifying cases in greater need of care (Dietch et al., 2021; Zhuang et al., 2023). Taken together, these findings suggest that the NDI not only effectively discriminates between different degrees of impairment, but also provides valuable information for clinical decision-making.

The implications of this research suggest both theoretical and practical contributions. Theoretically, the validation of the NDI in Spanish contributes to the development of psychometrics applied to the study of sleep disorders, offering a culturally adapted and sensitive instrument that advances the understanding of nightmares as a clinically relevant phenomenon. Practically, the NDI constitutes a valuable tool for mental health professionals, as it facilitates the early detection of problems associated with nightmares and allows for more precise referral to specialised services. In the adult population, this contribution is particularly relevant given the high comorbidity of nightmares with mental health problems (Sheaves et al., 2023). Its systematic use in clinical and community contexts could favour the identification of at-risk adults, including those who do not actively seek care but nevertheless experience significant deterioration in psychological wellbeing and quality of life (Etindele Sosso et al., 2023). In the Peruvian context, where psychometric studies on sleep are limited and mental health gaps persist, the availability of a validated instrument such as the NDI opens the possibility of developing epidemiological and clinical research to more accurately estimate the prevalence and impact of nightmares in the general adult population. It could also serve as a basis for implementing prevention and intervention programmes aimed at adults in vulnerable situations, such as individuals with limited access to health services, low socioeconomic resources, or a history of trauma. In this way, the NDI not only contributes to the academic field but also offers a practical resource with direct implications for public health and the improvement of mental health care.

## 5. Limitations

Among the main strengths of the present study are the use of advanced methods such as the Bayesian approach for estimating reliability, as well as the application of item response theory, which provides more robust evidence on item functioning and instrument precision. Nevertheless, some limitations should be acknowledged. Firstly, the sampling method was non-probabilistic, which limits the generalisability of the findings to other developmental stages such as adolescence or older adulthood. In addition, it is important to acknowledge sample biases, such as a higher proportion of women, participants residing in urban areas, and data collection conducted online, which may generate biases in the representation of the general population. Future research should strive for greater homogeneity in terms of sex, area of residence, and collection methods (face-to-face or mixed) to reduce potential biases. Test–retest reliability was not assessed due to the cross-sectional nature of the data; future studies could consider longitudinal data to examine the temporal stability of the NDI. Finally, in the analysis of convergent validity, variables related to nightmares were included. However, it is necessary for future studies to consider predictive validity. For example, testing whether NDI scores predict diagnostic status, treatment outcomes, or longitudinal changes in mental health. This would more strongly support the practical utility of the instrument, especially in health care and public health contexts.

## 6. Conclusions

In summary, the results of this study support the validity and reliability of the Spanish version of the NDI for the assessment of nightmares in adults from the general population. The scale showed a unidimensional structure, adequate reliability, measurement invariance by sex, and significant relationships with depressive symptoms, generalised anxiety, and suicidal ideation. In addition, item response theory provided evidence of solid performance, with particular precision at higher levels of nightmares. These findings suggest that the NDI is a useful tool for both research and clinical practice, especially in mental health contexts in Peru and other Spanish-speaking countries.

## Figures and Tables

**Table 1:** Final Spanish version of the NDI.

Items	Original Version in English	Spanish Version
1	How many nights a week did you have nightmares (i.e., disturbing, extended, well-remembered dreams)?	¿Cuántas noches por semana tuvo pesadillas (es decir, sueños perturbadores, prolongados y bien recordados)?
2	How often do you wake up from your nightmares and quickly become alert?	¿Con qué frecuencia se despierta de sus pesadillas y se pone alerta de manera rápida?
3	To what extent have nightmares troubled/distressed you in general?	¿En qué medida las pesadillas le han preocupado o angustiado en general?
4	To what extent have nightmares caused difficulties in social, work, or other areas of your life?	¿En qué medida las pesadillas han causado dificultades en el ámbito social, laboral u otras áreas de su vida?
5	How long have you been bothered by nightmares?	¿Cuánto tiempo lleva sufriendo pesadillas?

**Table 2:** Content validity of the NDI items.

Items	Relevance (n = 5)		Representativeness (n = 5)		Clarity (n = 5)	
	*V*	*CI* 95%	*V*	*CI* 95%	*V*	*CI* 95%
1	0.73	0.52, 0.87	0.93	0.75, 0.99	0.80	0.59, 0.92
2	0.87	0.67, 0.95	0.87	0.67, 0.95	0.80	0.59, 0.92
3	0.93	0.75, 0.99	0.73	0.52, 0.87	0.73	0.52, 0.87
4	0.87	0.67, 0.95	0.93	0.75, 0.99	0.87	0.67, 0.95
5	0.80	0.59, 0.92	0.87	0.67, 0.95	0.93	0.75, 0.99

Note. V = Aiken’s V, CI = confidence intervals.

**Table 3:** Descriptive measures and correlation matrix.

Item	*M*	*SD*	*g*_1_	*g*_2_	*r_it_*	Correlation Matrix				
1	0.61	0.79	1.19	1.02	0.68	-				
2	0.94	1.01	1.06	0.72	0.75	0.75	-			
3	0.80	0.96	1.25	1.19	0.73	0.72	0.82	-		
4	0.43	0.78	1.91	3.38	0.66	0.69	0.70	0.82	-	
5	0.64	1.27	1.85	1.95	0.49	0.61	0.65	0.56	0.56	-

Note. M = media, SD = standard deviation, g_1_ = skewness, g_2_ = kurtosis, *r*_it_ = corrected item test correlation.

**Table 4:** Invariance analysis of the NDI by sex.

Models	*X*^2^ (*df*)	*p*	*CFI*	*TLI*	*RSMR*	∆*CFI*	∆*TLI*	∆*RSMR*
M1	47.05 (10)	0.000	0.992	0.985	0.040	-	-	-
M2	51.79 (20)	0.000	0.993	0.993	0.040	0.001	0.009	0.000
M3	50.99 (24)	0.001	0.994	0.995	0.040	0.001	0.002	0.000
M4	56.15 (28)	0.001	0.994	0.996	0.041	0.000	0.000	0.001
M5	61.51 (33)	0.002	0.994	0.996	0.046	0.000	0.001	0.004

Note. M1 = configural invariance, M2 = threshold invariance, M3 = metric invariance, M4 = scalar invariance, M5 = strict invariance.

**Table 5:** Item discrimination, difficulty, and fit parameters.

Items	Item Parameters					Item Fit	
	*a*	*β*_1_	*β*_2_	*β*_3_	*β*_4_	*S-x^2^* (*df*)	*RMSEA.S-x* ^2^
1	2.73	0.196	1.291	2.380	3.099	10.28 (10)	0.007
2	3.65	−0.257	0.824	1.553	2.047	14.49 (19)	0.000
3	4.33	−0.026	0.968	1.580	2.106	19.04 (14)	0.027
4	3.30	0.672	1.345	2.172	2.755	24.91 (16)	0.033
5	1.73	0.988	1.398	1.745	1.931	42.18 (28)	0.032

Note. *a* = discrimination parameter. β = difficulty parameter. S-x^2^ = fit index. df = degrees of freedom. RMSEA.S-x^2^ = root mean square error of approximation.

## Data Availability

The data presented in this study are available upon request from the corresponding author due to privacy issues.
